# Influence of the active second stage of labor in nulliparous women on umbilical cord blood pH and neonatal outcomes: a population-based, cohort study

**DOI:** 10.1186/s12884-025-07917-1

**Published:** 2025-08-08

**Authors:** Lisa Holmberg, Linda Iorizzo, Mehreen Zaigham

**Affiliations:** 1 Department of Clinical Sciences Lund, Lund University, Lund, Sweden; 2 Department of Obstetrics and Gynaecology, Skane University Hospital Lund, Lund, Sweden; 3 Department of Obstetrics and Gynaecology, Skane University Hospital Malmo, Malmo, Sweden; 4 Department of Obstetrics & Gynecology, Lund University and Skåne University Hospital Lund, Lund, 221 85 Sweden

**Keywords:** Acidosis, Birth, Labor, Neonatal outcomes, Perinatal asphyxia, Second stage, Umbilical cord blood pH

## Abstract

**Background:**

Prolonged second stage of labor increases the risk of insufficient gas exchange over the placenta to the fetus and risk of birth asphyxia. There are considerable variations in clinical guidelines regarding the recommended duration of pushing during the active second stage of labor. Thus, the aim of this study was to investigate the total duration of the second stage of labor and its association to changes in umbilical cord pH and adverse neonatal outcomes in nulliparous women.

**Methods:**

This retrospective, multi-center cohort study was based on data from the Perinatal Revision South Register covering seven maternity units from 1995 to 2015. Nulliparous women with fetuses in cephalic position and complete and validated cord blood pH data were recruited to the study. Logistic regression was used to establish the relationship of duration of pushing, categorized in 60 min increments, with cord blood acidosis (umbilical cord arterial pH < 7.05) and adverse neonatal outcomes, including Apgar score at 5 min, risk for central nervous system (CNS) disease, utilization of continuous positive airway pressure (CPAP), neonatal intensive care unit (NICU) admission and hypoxic ischemic encephalopathy (HIE). The adjusted model adjusted for maternal BMI, gestational duration and birth weight.

**Results:**

A total of 37,008 women were included in the analysis. For every 60 min increase in pushing time, there was a significantly increased adjusted odds ratio (aOR) for umbilical arterial pH < 7.05, aOR = 1.639, (95% CI = 1.418–1.895, *P* = 0.02), Apgar score < 7 at 5-minutes, aOR = 1.408 (95% CI = 1.082–1.831, *P* = 0.01), and prevalence of suspected CNS disease, aOR = 1.417, (95% CI = 1.065–1.886, *P* = 0.02). The use of CPAP, (aOR = 1.215, 95% CI = 0.945–1.561, *P* = 0.13), NICU admission (aOR = 1.021, 95% CI = 0.890–1.171, *P* = 0.77), neonatal seizures (aOR = 0.000, *P* = 0.98) and HIE (aOR = 1.377, 95% CI = 0.416–4.109, *P* = 0.57) were not associated with increased pushing time.

**Conclusions:**

Using a large, population-based cohort, we found that prolonged active second stage of labor was associated with an increased odds for cord blood acidosis at birth, low Apgar score at 5 min, and suspected CNS-disease in neonates. Efficient and evidence-based time management during the active second stage of labor is therefore crucial for reducing the risk of adverse neonatal outcomes.

**Supplementary Information:**

The online version contains supplementary material available at 10.1186/s12884-025-07917-1.

## KEY MESSAGE

Prolonged active second stage of labor increases the risk of cord blood acidosis at birth, low Apgar scores, and neonatal CNS disease. Effective, evidence-based time management is essential to minimize these risks and improve neonatal outcomes in clinical practice.

**Supplementary Information:**

The online version contains supplementary material available at 10.1186/s12884-025-07917-1.

## Background

One of the leading causes of neonatal mortality and morbidity is perinatal asphyxia [1]. In 2017, the World Health Organization (WHO) documented 610,000 global fatalities due to perinatal asphyxia and trauma, constituting 24% of all deaths in neonates aged 27 days or younger [2].

The second stage of labor is when the cervix is fully dilated and is divided into the ‘passive’ and the ‘active’ phases. The ‘active phase of the second stage of labor’ is when the parturient initiates active pushing. The duration of the parturient ´s pushing efforts plays a crucial role in determining the risk of developing perinatal asphyxia since an extended active second stage of labor increases the risk of insufficient oxygen supply to the fetus [3, 4]. There are considerable variations in guidelines and clinical recommendations regarding the duration of pushing during the active second stage of labor [5–7]. Optimizing the timing of the active part of the second stage of labor could improve labor management and thus prevent perinatal asphyxia.

Previous studies regarding the duration of the second stage of labor show conflicting results. Some authors found no correlation between neonatal outcomes and pushing time [8–16] while other studies have found that a prolonged second stage increases the risk of low 5-min Apgar-score, admission to neonatal intensive care units (NICUs), prolonged hospital stays, neonatal sepsis and increased overall perinatal morbidity [17–22]. A recent systematic review concluded that a prolonged duration of second stage of labor increased the risk of neonatal complications both in nulliparous and multiparous women [23]. Regarding the active phase of the second stage of labor, literature is more limited. Sandström et al. [22] and Grobman et al. [24] found that an extended second stage of labor was associated with an increased risk of composite adverse neonatal outcomes, and individually with brachial plexus palsy, clavicular fracture, skull fracture, seizures, HIE, umbilical artery acidosis (defined as pH < 7.05 and base excess < −12 mmol/L) and admission to NICU. Both studies showed a significant correlation between pushing time and composite adverse neonatal outcomes, however the incidence of the events were rare and thus had a small absolute risk difference. Other studies have shown that a prolonged duration of second stage of labor is directly associated with higher levels of metabolites indicating fetal hypoxia, including lactic acid, carbon dioxide and lower pH in umbilical cord blood gases [22, 25–28]. Contradicting these findings, some studies have concluded that there is no discernible link between extended pushing and adverse neonatal outcomes or alterations in umbilical cord arterial pH (UApH) levels [28, 29].

Therefore, using a large population-based cohort, we aimed to investigate the correlation between the duration of the specific active second stage of labor, to alterations in umbilical cord arterial pH and adverse neonatal outcomes such as Apgar score < 7 at 5-minutes, need for CPAP, NICU-admission, seizures, hypoxic ischemic encephalopathy and CNS-disease in nulliparous women.

## Methods

This multi-center, retrospective cohort study was conducted from 1995 to 2015 in the South of Sweden. Data on maternal and neonatal characteristics, including birth data, was retrieved from the Perinatal Revision South Register (PRSR). The PRSR is a quality database with data from nine maternity units in the Regions of Skåne, Halland, Kronoberg and Blekinge. We included all cases of nulliparous women with intended vaginal labor, singleton term fetus, in cephalic presentation. Cases with intended vaginal labor but delivered by emergency section were also included as well as women with pregnancy complications.

The study was approved by the Swedish Ethical Review Authority and the requirement for informed consent was waived by Regional Ethics Committee in Lund due to the retrospective and population-based design of the study.

**Fig. 1:**
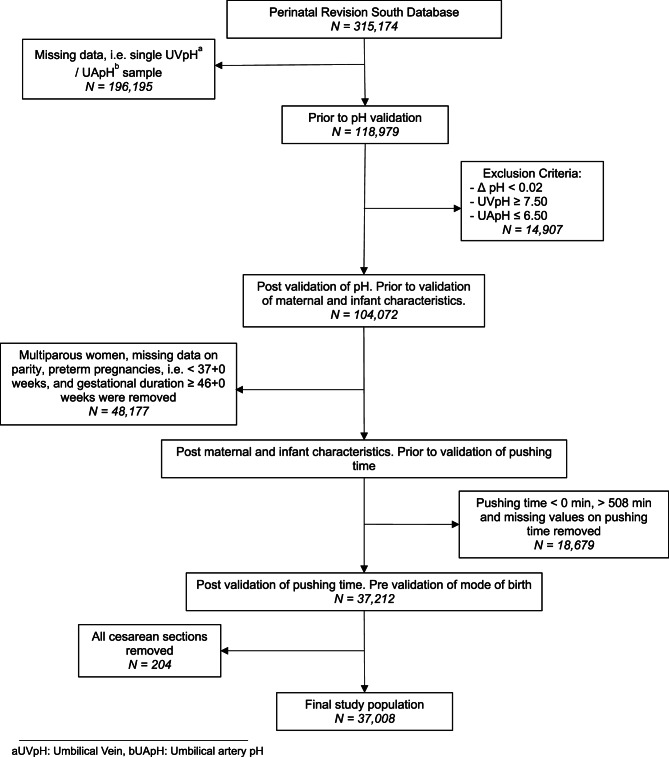
Flowchart of the study population. Presented with exclusion criteria for validation of pH, pushing time and maternal, labor and infant characteristics

The original dataset consisted of 315,174 births before validation of cord blood pH values (Fig. 1). Cases with missing data on either umbilical venous pH (UVpH) or umbilical artery pH (UApH) were excluded. Samples with a veno-arterial difference of ≤ 0.02 were excluded to ensure inclusion of samples taken from different vessels. Additionally, outliers with an UVpH of more than 7.50 or an UApH of less than 6.50 were removed since they were implausible or likely recording errors. Likewise, patients with negative value of pushing time, missing data on pushing time and pushing time that extended 508 min (99.9th percentile) were also excluded.

Duration of pushing was categorized into 60 min increments, resulting in four groups: 0 to 60 min, 61 to 120 min, 121 to 180 min and > 180 min.

### Outcomes

Neonatal outcome variables included UApH, Apgar score at 5-minutes, use of continuous positive airway pressure (CPAP), NICU admission, neonatal seizures, hypoxic ischemic encephalopathy (HIE) and central nervous system (CNS) disease. According to the clinical guidelines paired umbilical cord blood samples were collected from intact cord on all newborns directly at birth and analyzed within 15 min. Umbilical artery acidosis was defined as umbilical artery pH value less than 7.05 [30, 31]. CNS disease was defined as any suspected brain injury at birth that required further specialized examination and testing for example hypotonia, seizures, lethargy or altered mental state.

Gestational duration was subdivided into three groups: full-term I (37 + 0–40 + 6 weeks), full-term II (41 + 0–41 + 6 weeks), and post term (≥ 42 weeks).

### Statistical analysis

Demographic data was described as absolute numbers and percentages. Changes in average pushing time were further investigated using six categories of UApH values < 7.000, 7.000-7.049, 7.050–7.099, 7.100-7.149, 7.150–7.199 and ≥ 7.200. Logistic regression was used to investigate the relationship between pushing time, as both a categorical and continuous variable (Supplementary Table 1), to neonatal outcomes as dichotomous variables. The model was adjusted for maternal BMI, gestational age, and neonatal birth weight. Results were presented as crude and adjusted odds ratios (OR) for the outcome variables with 95% confidence intervals. A two-tailed *P*-value < 0.05 was considered statistically significant. Statistical analyses were conducted using SPSS (29.0) (IBM Corp, Armonk, NY) and STATA (StataCorp. 2023. Stata Statistical Software: Release 18. College Station, TX: StataCorp LLC).

## Results

**Fig. 2:**
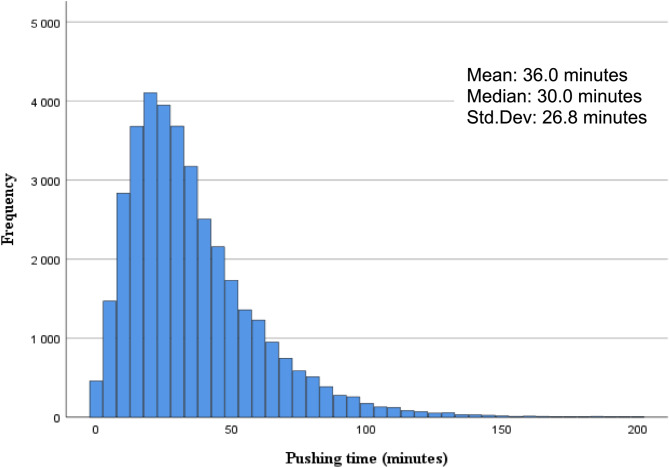
Histogram of distribution of pushing time in the study population. X-axis representing pushing time in minutes and y-axis the number of individuals. Median pushing time being 30 min. *N* = 37,*008*

A total of 37,008 nulliparous parturients met the inclusion criteria for the final analysis. Demographic data is presented in Table 1. The median duration of pushing was 30 min and 95% of the parturients gave birth within 83 min of active pushing (Lower Quartile 19 min; Upper Quartile 47 min). (Fig. 2). Median pushing time was shorter for the categories in the normal range of UApH values. For a UApH ≥ 7.20 the median pushing time was 28 min (Table 2**)**. The median pushing time increased for lower UApH classes, with median pushing time of 41 min in the acidotic, UApH < 7.05 category.

**Table 1:** Demographic characteristics of the study population

		*N*	%
Total N		37,008	100.0
Maternal age (years)			
	*< 20*	1,397	3.8
	*20–34*	32,005	86.5
	*35–39*	3,163	8.5
	*≥ 40*	443	1.2
Maternal BMI ^a^			
	*< 18*,*5*	639	1.7
	*18.5–24.9*	13,871	37.5
	*25-29.9*	5,044	13.6
	*≥ 30*	2,060	5.6
	*Missing data*	15,394	41.6
Maternal smoking			
	*No*	23,440	63.3
	*Yes*,* 1–9/day*	1,627	4.4
	*Yes*,* ≥ 10/day*	442	1.2
	*Missing data*	11,499	31.1
Mode of birth start			
	*Spontaneous*	33,578	90.7
	*Induction*	3,425	9.3
	*Missing data*	5	0.0
Birth mode			
	*Vaginal*,* non-instrumental*	31,626	85.5
	*Vaginal*,* VE* ^*b*^*/forceps*	4,856	13.1
	*Missing data*	526	1.4
Gestational duration in weeks			
	*37 + 0–40 + 6*	28,117	76.0
	*41 + 0–41 + 6*	6,676	18.0
	*≥ 42 + 0*	2,215	6.0
Gender			
	*Male*	18,954	51.2
	*Female*	18,021	48.7
	*Unknown*	33	0.1
Birth weight (grams)			
	*< 2500*	290	0.8
	*2500–3999*	31,225	84.4
	*4000–4499*	4,662	12.6
	*≥ 4500*	816	2.2
	*Missing data*	15	0.0
Total duration of pushing time			
	*0–60 min*	31,910	86.2
	*61–120 min*	4,712	12.7
	*121–180 min*	296	0.8
	*> 180 min*	89	0.2
Apgar Score at 5-minutes			
	*0–3*	53	0.1
	*4–6*	292	0.8
	*7–10*	36,658	99.1
	*Missing data*	5	0.0
^a^*BMI,* Body Mass Index, ^b^*VE*, Vacuum Extraction			

**Table 2:** Percentile distribution of pushing time in relation to umbilical arterial pH groups. *N* = 37,008

Pushing time (minutes)									
UApH ^a^	*N*	*5*	*10*	*25*	*Median*	*75*	*90*	*95*	*Mean*
< 7.000	373	9.7	12.4	24.5	41.0	59.0	91.8	109.0	47.2
7.000-7.049	734	9.0	14.0	22.0	37.0	59.0	80.5	97.3	44.0
7.050–7.099	1,846	9.0	13.0	22.0	36.0	53.0	75.0	90.0	41.0
7.100-7.149	4,299	10.0	14.0	22.0	34.0	53.0	73.0	87.0	40.3
7.150–7.199	7,480	8.0	12.0	20.0	32.0	50.0	71.0	86.0	38.2
≥ 7.200	22,276	6.0	10.0	17.0	28.0	44.0	64.0	79.0	33.3
^a^*UApH*, Umbilical Artery pH									

Among the neonates, a total of 3.0% (*n* = 1,107) had an UApH less than 7.05, 0.9% (*n* = 345) had an Apgar Score < 7 at 5-minutes, 1.3% (*n* = 468) needed CPAP, 6.4% (*n* = 2,384) were admitted to NICU, 0.1% (*n* = 47) experienced seizures and 0.8% (*n* = 281) had suspected CNS disease and 0.1% (*n* = 34) were diagnosed with HIE (Table 3).

**Table 3:** Odds ratio of adverse neonatal outcomes in relation to pushing time, categorized in 60 min increments. Logistic regression was used with 95% confidence intervals and *P*-values

		*N*	%	OR (95% CI)	*P*-value	Adjusted OR* (95% CI)	*P*-value
Total N		37,008	100				
UApH ^a^							
	*< 7.05*	1,107	3.0	1.720 (1.537–1.925)	< 0.001	1.639 (1.418–1.895)	< 0.001
	*≥ 7.05*	35,901	97.0	Ref		Ref	
Apgar score 5-minutes							
	*< 7*	345	0.9	1.344 (1.074–1.683)	0.010	1.408 (1.082–1.831)	0.011
	*≥ 7*	36,658	99.1	Ref		Ref	
CNS ^b^ Disease							
	*No history of CNS disease*	36,727	99.2	Ref		Ref	
	*CNS disease*	281	0.8	1.238 (0.954–1.608)	0.108	1.417 (1.065–1.886)	0.017
CPAP ^c^							
	*No*	36,540	98.7	Ref		Ref	
	*Yes*	468	1.3	1.355 (1.118–1.643)	0.002	1.215 (0.945–1.561)	0.129
Hypoxic ischemic encephalopathy							
	*No history of HIE* ^*d*^	36,974	99.9	Ref		Ref	
	*History of HIE*	34	0.1	0.978 (0.415–2.302)	0.959	1.377 (0.416–4.109)	0.566
NICU ^e^ Admission							
	*Not admitted to NICU*	34,624	93.6	Ref		Ref	
	*Admitted to NICU*	2,384	6.4	1.083 (0.979–1.196)	0.122	1.021 (0.890–1.171)	0.770
Neonatal seizures							
	*No history of seizures*	36,961	99.9	Ref		Ref	
	*History of seizures*	47	0.1	0.432 (0.140–1.335)	0.145	0.000 (0.000 -.)	0.982
*Adjusted for maternal BMI, gestational duration, and birth weight ^a^*UApH*, Umbilical Arterial pH, ^b^*CNS*, Central Nervous System, ^c^*CPAP*, Continuous Positive Airway Pressure, ^d^*HIE*, Hypoxic Ischemic Encephalopathy, ^e^*NICU*, Neonatal Intensive Care Unit							

Logistic regression analyses, indicated that for every 60 min increase in pushing time, there was an increase in crude OR for UApH < 7.05 (OR = 1.720, 95% CI = 1.537–1.925, *P = < 0.001*), Apgar score < 7 at 5-minutes (OR = 1.334, 95% CI = 1.074–1.683, *P* = 0.01) and need for CPAP (OR = 1.355, 95% CI = 1.118–1.643, *P = < 0.001*). There was no relationship between NICU admission, neonatal seizures, suspected CNS disease and HIE to increased duration of pushing time (Table 3**)**. When adjusted for maternal BMI, length of gestation and birth weight, UApH < 7.05 (aOR = 1.639, 95% CI = 1.418–1.895, *P = < 0.001*) and Apgar score < 7 at 5-minutes (aOR = 1.408, 95% CI = 1.082–1.831, *P* = 0.01) remained significant and prevalence of suspected CNS disease (aOR = 1.417, CI = 1.065–1.886, *P* = 0.02), now showed a correlation to increased duration of pushing time. For CPAP need, there was no increased risk, as was the case for NICU, neonatal seizures and HIE.

When pushing time was considered a continuous variable (Supplementary Table 1), every one minute increase in pushing time was associated with a slight increase in risk for UApH < 7.05 (OR = 1.008, 95% CI = 1.006–1.009, *P = < 0.001*), Apgar score < 7 at 5-minutes (OR = 1.006, 95% CI = 1.003–1.008, *P = < 0.001*) and the use of CPAP (OR = 1.007, 95% CI = 1.005-1,009, *P = < 0.001*). When we compared NICU admission, neonatal seizures, CNS disease and HIE with duration of pushing, we found no significant relationship. However, when adjusted for maternal BMI, gestational age and birth weight, the prevalence of CNS disease showed a slightly increased odds with every unit increase in pushing time (aOR = 1.005, 95%CI = 1.002–1.009, *P* = 0.007). An increased odds UApH < 7.05 (aOR = 1.006, 95%CI = 1.004–1.008, *P = < 0.001*), Apgar score < 7 at 5-minutes (aOR = 1.006, 95%CI = 1.003–1.009, *P = < 0.001*) and need for CPAP (aOR = 1.005, 95%CI = 1.003–1.008, *P = < 0.001*), with every unit increase in pushing time remained significant after adjustments.

## Discussion

This large, population-based cohort study investigated the relationship between the duration of active pushing in the second stage of labor in nulliparous to changes in UApH, as well as its impact on adverse neonatal outcomes. Our findings demonstrate that with each additional hour of pushing, there was an increased odds for neonatal acidosis, low Apgar score at 5 min and CNS disease. However, no significant relationship was found between increased duration of pushing and odds of need for CPAP, HIE, NICU admission, and neonatal seizures.

A large Swedish study by Sandström et al. with 42,539 nulliparous women, demonstrated that the rates of birth asphyxia related complications and admission to NICU, gradually increased with prolonged second stage [22]. In accordance with our findings, they also showed that acidosis (defined as pH < 7.05 and BE < −12 mmol/L) correlated with extended duration of pushing ≥ 60 min compared to ≤ 15 min, but in the current study no significant relationship between pushing time and admission to NICU was found. An American study by Grobman et al. [24] included both nulliparous and multiparous women, reported that a longer duration of pushing was associated with an increased OR of composite adverse neonatal outcome. Unlike Grobman et al. and Sandström et al., the present study examined neonatal outcomes separately, and not as a composite outcome, which enabled to study the relationship of outcomes individually to increasing pushing time [22, 24]. Additionally, the incidence of adverse outcomes was rare in both studies, and thus the authors reported small absolute risks of 1.29% respectively 3% [22, 24]. Rare outcomes are always a challenge to study and the present study could not find increased odds of HIE or neonatal seizures with prolonged pushing time.

The lack of significance may reflect small numbers and should not be interpreted as that there is no risk.

In accordance with the present study, Yli B et al. investigated 40,000 nulliparous and multiparous women in Europe and found a significant increased risk of neonates with Apgar score < 7 at 5-minutes, when the duration of active second stage of labor exceeded 60 min [27]. Furthermore, they also demonstrate that, already after 15 min of pushing, there was a significantly increased probability of the fetus developing metabolic acidosis. Le Ray et al. concluded that, after the first hour of expulsive efforts, the probability of spontaneous birth of a neonate who had no indicators of asphyxia decreased with time of pushing [29]. In contrast, a recent but smaller study involving 6804 nulliparous women, concluded that there were no differences between an active second stage of labor of ≤ 60 min and > 60 min concerning low Apgar scores < 7 at 5-minutes and admission to NICU (within 24 h) [28].

Managing a prolonged second stage of labor requires clinical skills and judgement. Implementing evidence-based strategies in handling the second stage of labor plays a crucial role in the improvement of obstetric care, guiding informed decisions and optimizing outcomes. The association between pushing time and adverse neonatal outcomes is not fully understood and previous research on the subject has not reached a consensus. Acidemia, or a sustained lack of oxygen at birth could play a major role. The findings of this study, with an increased OR for UApH < 7.05 with prolonged duration of the active second stage of labor, is in accordance with previous studies that have concluded that a longer duration of the second stage of labor is associated with an increased risk of CTG abnormalities, severe acidemia, metabolic acidosis and higher concentrations of lactic acid [25, 27, 28, 32]. In a recently published cohort study of almost 36,000 Swedish neonates, the long-term outcomes of being born with acidemia (UApH < 7.05) were investigated with a follow-up time up to 20 years of age. The findings show a UApH < 7.05 to be associated with not only an increased risk of death, but also to neurodevelopmental disorders like cerebral palsy and epilepsy [31]. These findings underscore the critical importance of good obstetric management of the active second stage of labor.

It is an ongoing challenge to optimize obstetric management and the definition of prolonged second stage of labor which has undergone changes in recent years. The WHO recommends that when the second stage of labor exceeds 3 h in nulliparous women and 2 h in multiparous women, obstetric healthcare providers should consider intervention to expedite birth since the likelihood of spontaneous birth decreases [3]. In Sweden, guidelines suggest that if there is a 2–3 h delay in expected cervical dilation during the first stage of labor, it is advisable to consider expediting childbirth, firstly by amniotomy and then by oxytocin stimulation [33]. However, guidelines concerning active pushing time are sparse and, to the best of our knowledge, a global consensus on recommended time of pushing does not currently exist. Only recently The American College of Obstetricians and Gynecologists (ACOG) published a clinical practice guideline for First and Second Stage Labor Management [7]. They strongly recommend that pushing commence as soon as complete cervical dilation is achieved and that prolonged second stage of labor be defined as more than 3 h of pushing in nulliparous and 2 h of pushing in multiparous women [7]. From our results, to allow three hours of pushing in nulliparous is questionable since it may impose an increased risk for hypoxia in the fetus. As hypoxia is a major contributor to neonatal morbidity and mortality, optimal timing of the active part of the second stage of labor can allow for improved labor management and prevention of perinatal asphyxia. The ultimate goal is individualized intrapartum care based on a comprehensive evaluation of multiple risk factors where prolonged time in second stage requires specific consideration and optimized care to balance the negative consequences of interventions and the risk for the fetus with continued labor.

### Strengths and limitations

The large cohort size, including data on pushing time from over 35,000 births was a major strength of the current study. This enabled comprehensive analysis of individual outcomes, even though these outcomes are rare in Sweden [34], including adjustment for possible confounders, allowing assessment of pushing time in the context of multiple obstetric covariates. Strict validation of the umbilical cord blood values was also performed, removing all cases where data was not validated, which enabled a comprehensive study of the alterations in UApH across pushing times. In addition, blood gas samples are routinely collected in the Swedish birth units which ensures good quality of the samples included in the study [35]. However, if missing values for pH were more common among the most depressed neonates, this may contribute to selection bias and potentially underestimation of the association between duration of pushing and the risk adverse neonatal outcomes. On the other hand, if a neonate is vigorous directly after birth, blood gas analysis may not be prioritized by healthcare providers and data from these cases may also be missing.

Regression analyses were adjusted for maternal BMI, gestational age and birth weight. However, it was not possible to stratify analyses based on the use of epidural analgesia and use of oxytocin since this information was not included in the birth register used. These factors may affect pushing time, either by prolonging or speeding up birth, and thus pose as possible confounders. For neonatal seizures, only a lower limit of CI was recorded in the statistical analysis which is not an uncommon occurrence when performing multivariate logistic regression. Possible reasons could be due to a low incidence of seizures overall in our study population or because of the number of categories we divided pushing time into. An additional limitation involves diagnosing CNS disease based on clinical signs and defined as suspected injury. Due to this definition, there’s a potential for overdiagnosis, possibly amplifying the perceived association.

## Conclusions

Prolonged duration of the active second stage of labor > 60 min in nulliparous women contributed to an increased risk of cord blood acidosis and low Apgar score at five minutes in a high resource setting. Apart from suspected CNS-disease, we found no increased risk for other long term adverse neonatal outcomes. Further studies, in larger more diverse populations and settings are indicated to delineate specific neonatal outcomes further.

## Supplementary Information

Supplementary Material 1. Table 1. Logistic regression of outcome variables in relation to pushing time as a continuous variable with one minute as the unit of time.

## Data Availability

The datasets generated and analyzed during the current study are not publicly available due to agreements signed with the Regional Ethics Committee in Lund under the Ethical Approval of the study but are available from the corresponding author on reasonable request.

## Abbreviations

aOR: Adjusted Odds Ratio
NICU: Neonatal Intensive Care Unit
PRSR: Perinatal Revision South Register
UApH: Umbilical Cord Arterial pH
UVpH: Umbilical Cord Venous pH
