# Supplementary material for: Influence of the active second stage of labor in nulliparous women on umbilical cord blood pH and neonatal outcomes: a population-based, cohort study
Source: BMC Pregnancy Childbirth. 2025 Aug 8;25:825. doi: 10.1186/s12884-025-07917-1 (PMC12333123; doi:10.1186/s12884-025-07917-1)
Supplement: Supplementary file 1 — Supplementary Material 1. Table 1. Logistic regression of outcome variables in relation to pushing time as a continuous variable with one minute as the unit of time. [file 12884_2025_7917_MOESM1_ESM.docx]

| **Supplementary Table 1.**  Logistic regression of outcome variables in relation to pushing time as a continuous variable with one minute as the unit of time. | | | | | | | |
| --- | --- | --- | --- | --- | --- | --- | --- |
|  |  | ***N*** | ***%*** | ***OR (95% CI)*** | ***P*** | ***Adjusted OR* (95% CI)*** | ***P*** |
| **Total N** | | 37,008 | 100 |  |  |  |  |
| **UApH** ^a^ | |  |  |  |  |  |  |
|  | *< 7.05* | 1,107 | 3.0 | **1.008 (1.006-1.009)** | **<0.001** | **1.006 (1.004-1.008)** | **<0.001** |
|  | *≥ 7.05* | 35,901 | 97.0 | Ref |  | Ref |  |
| **Apgar Score 5-minutes** | |  |  |  |  |  |  |
|  | *< 7* | 345 | 0.9 | **1.006 (1.003-1.008)** | **<0.001** | **1.006 (1.003-1.009)** | **<0.001** |
|  | *≥ 7* | 36,658 | 99.1 | Ref |  | Ref |  |
| **CNS** ^b^ **Disease** | |  |  |  |  |  |  |
|  | *No history of CNS disease* | 36,727 | 99.2 | Ref |  | Ref |  |
|  | *CNS disease* | 281 | 0.8 | 1.002 (0.998-1.006) | 0.239 | **1.005 (1.002-1.009)** | **0.007** |
| **CPAP** ^c^ | |  |  |  |  |  |  |
|  | *No* | 36,540 | 98.7 | Ref |  | Ref |  |
|  | *Yes* | 468 | 1.3 | **1.007 (1.005-1.009)** | **<0.001** | **1.005 (1.003-1.008)** | **<0.001** |
| **Hypoxic ischemic encephalopathy** | |  |  |  |  |  |  |
|  | *No history of HIE* ^d^ | 36,974 | 99.9 | Ref |  | Ref |  |
|  | *History of HIE* | 34 | 0.1 | 1.004 (0.994-1.014) | 0.438 | 1.006 (0.996-1.017) | 0.227 |
| **NICU** ^e^ **Admission** | |  |  |  |  |  |  |
|  | *Not admitted to NICU* | 34,624 | 93.6 | Ref |  | Ref |  |
|  | *Admitted to NICU* | 2,384 | 6.4 | 1.001 (1.000-1.003) | 0.066 | 1.002 (1.000-1.004) | 0.043 |
| **Neonatal seizures** | |  |  |  |  |  |  |
|  | *No history of seizures* | 36,961 | 99.9 | Ref |  | Ref |  |
|  | *History of seizures* | 47 | 0.1 | 0.993 (0.980-1.006) | 0.263 | 0.979 (0.958-1.001) | 0.067 |
| *Adjusted for maternal BMI, gestational duration, and birth weight  ^a^UApH: Umbilical Arterial pH, ^b^CNS: Central Nervous System, ^c^CPAP: Continuous Positive Airway Pressure, ^d^HIE: Hypoxic Ischemic Encephalopathy, ^e^NICU: Neonatal Intensive Care Unit. | | | | | | | |
